# Social environment during egg laying: Changes in plasma hormones with no consequences for yolk hormones or fecundity in female Japanese quail, *Coturnix japonica*

**DOI:** 10.1371/journal.pone.0176146

**Published:** 2017-05-03

**Authors:** Esther M. A. Langen, Nikolaus von Engelhardt, Vivian C. Goerlich-Jansson

**Affiliations:** 1 Department of Animal Behaviour, Bielefeld University, Bielefeld, Germany; 2 Department of Animals in Science and Society, Utrecht University, Utrecht, The Netherlands; 3 Faculty of Science and Engineering, University of Plymouth, Plymouth, United Kingdom; University of Vienna, AUSTRIA

## Abstract

The social environment can have profound effects on an individual’s physiology and behaviour and on the transfer of resources to the next generation, with potential consequences for fecundity and reproduction. However, few studies investigate all of these aspects at once. The present study housed female Japanese quail (*Coturnix japonica*) in pairs or groups to examine the effects on hormone concentrations in plasma and yolk and on reproductive performance. Circulating levels of androgens (testosterone and 5-α-dihydrotestosterone) and corticosterone were measured in baseline samples and after standardised challenges to assess the responsiveness of the females’ endocrine axes. Effects of the social environment on female fecundity were analysed by measuring egg production, egg mass, fertilization rates, and number of hatched offspring. Counter to expectation, females housed in pairs had higher plasma androgen concentrations and slightly higher corticosterone concentrations than females housed in groups, although the latter was not statistically significant. Pair vs. group housing did not affect the females’ hormonal response to standardised challenges or yolk testosterone levels. In contrast to previous studies, the females’ androgen response to a gonadotropin-releasing hormone challenge was not related to yolk testosterone levels. Non-significant trends emerged for pair-housed females to have higher egg-laying rates and higher fertility, but no differences arose in egg weight or in the number, weight or size of hatchlings. We propose that our unexpected findings are due to differences in the adult sex ratio in our social treatments. In pairs, the male may stimulate female circulating hormone levels more strongly than in groups where effects are diluted due to the presence of several females. Future studies should vary both group size and sex composition to disentangle the significance of sexual, competitive and affiliative social interactions for circulating and yolk hormone levels, and their consequences for subsequent generations.

## Introduction

The social environment of an individual can profoundly affect its behaviour, morphology and physiology. In many vertebrates, including birds, the frequency and type of social interactions affect circulating androgen and glucocorticoid levels [1–3]. Social interactions influence steroid hormones which, in turn, affect social and reproductive behaviour [4, 5]. Hence steroid hormones can act as mediators between the social environment and behaviour [1, 6], which ultimately can affect survival and reproduction [7, 8]. During reproduction, the social environment not only affects the individual itself, but also the amount of resources and other substances transferred to the next generation, potentially affecting offspring fitness [9, 10]. Such socially induced maternal effects enable parents to prepare offspring for their future social conditions, potentially resulting in adaptive transgenerational plasticity [11–14]; but see [15]. The mechanisms underlying the effects of the social environment on female physiology and behaviour and the consequences for fecundity and offspring quality are not yet well understood and deserve further research.

Gonadal steroids, regulated by the hypothalamic-pituitary-gonadal (HPG) axis, are important mediators of social interactions, especially in a reproductive context. Androgens, in particular testosterone (T), are involved in social interactions such as competition and aggression, as well as reproductive behaviour and physiology [1, 3]. In birds, female plasma androgen levels have been found to be positively correlated with conspecific competition and breeding density [16–20]. The link between plasma androgen levels and intra-sexual competition has been extensively studied under the “challenge hypothesis” [21], which states that, during reproduction, plasma T correlates positively with male-male competition. In females, although there are fewer studies than in males, similar hormonal responses to social challenges have been observed [16, 18, 22, 23], yet studies have also reported no link, or even negative correlations between female-female competition and circulating plasma androgen levels [19, 24–27]. Given these contradictory findings, further research is required to clarify the relationship between intra-sexual competition and circulating androgens in females.

Next to gonadal steroids, glucocorticoids play an important role in social behaviour. In avian species, corticosterone (CORT) is typically released under metabolic or otherwise challenging conditions, through activation of the hypothalamic-pituitary-adrenal (HPA) axis, and is therefore often referred to as a ‘stress hormone’. Socially challenging interactions can stimulate the HPA axis and increase circulating glucocorticoid levels, while affiliative social interactions can buffer the response to stressors [2, 28]. In birds, social density and circulating baseline CORT concentrations frequently are positively correlated [29–32]; but see [32–34]. In Japanese quail (*Coturnix japonica*), females housed in unstable social environments have higher plasma CORT concentrations following changes to the social environment compared to females kept in stable social environments [35]. Moreover, social interactions between Japanese quail females and an unfamiliar conspecific result in elevated CORT levels [36].

Circulating levels of androgens and CORT can affect female behaviour and reproductive investment, thereby influencing reproductive success both positively and negatively. Artificially elevated female plasma androgens have been shown to negatively affect reproduction [37–41], although the long-term effects on lifetime reproductive success may be small [39]. However, circulating androgens may have indirect positive effects on female reproductive success, for example by affecting competition, mate and nest acquisition and parental behaviour [16, 22, 42–44]. Circulating female CORT levels have been found to both negatively [45–50] and positively correlate with reproduction [48–51]. In Japanese quail, selection lines bred for an exaggerated stress response showed a decrease in reproductive success, with an additional negative effect of artificially increasing CORT levels in these females [52]. It is still unclear what causes the variable effects of androgens and CORT on reproduction. Possible explanations include context-dependent effects, time-dependent effects and non-linear effects of increasing hormone concentrations [48, 49, 53, 54].

In reproducing female birds, not only are plasma levels of steroids affected by the social environment, but also the deposition of hormones into yolk of developing eggs [10, 55, 56]. Breeding density and female-female competition is positively correlated with yolk androgens in many bird species [17, 57–62]. In the Japanese quail, yolk androgens are increased by social instability [35] and by selection for a high motivation to reinstate social contact [63]. The relationship between female plasma androgen levels and yolk androgen levels is still unclear [64, 65], but recent studies have suggested that variation in yolk hormone levels reflects differences in HPG axis sensitivity. Indeed, the increase of circulating androgens in response to gonadotropin-releasing hormone injections (GnRH) correlates positively with yolk androgen deposition in some bird species [66, 67], including Japanese quail [68]. This suggests a link between the social environment, the plasma androgen response to GnRH and yolk androgen levels.

Steroid hormones in the yolk influence the development and behaviour of offspring and are therefore important mediators of prenatal maternal effects. Yolk androgens influence fundamental traits such as offspring growth (both pre-and post-natal), timing of hatching, offspring immunity and behaviour [10, 69]. These factors can have consequences for offspring survival thus ultimately affecting the parents’ reproductive success [10, 69].

Given the contradictory findings on the relation between social stimulation, plasma androgens, CORT and yolk androgens, and their effects on reproduction, we explored the effects of the social environment in captive housed female Japanese quail. We kept the birds either in pairs (one male and one female) or in small groups of three females with one male to represent variation in the social system during breeding which may be found in the wild and in captivity. Japanese quail have been described as (serially) monogamous, polygynous, and polyandrous [70–72]. Studies on domesticated Japanese quail have shown that formation of (temporary) pair bonds indeed occurs, but the frequency of extra-pair copulations is high, and under laboratory conditions this species is usually housed in polygynous groups [70–73]. Overall, this suggests that the mating system is flexible in Japanese quail, which makes this a suitable species to study the effects of variation in the social environment on physiology and behaviour [70, 72, 74].

Compared to pair housing, group living should allow for more social interaction and potentially increase competition for resources and the male mating partner. These social stimulations are expected to result in changes in plasma and yolk hormone levels. We analysed the effect of social housing conditions on female plasma androgen and corticosterone levels and yolk T concentrations in their eggs. We refer to plasma androgens rather than T because the assay used cross-reacted to 23.3% with 5-α-dihydrotestosterone (5-α-DHT; see Methods), a potent androgen present in avian plasma [75–77]. To investigate the effects of the social environment on HPG and HPA axis sensitivity, we tested the females’ physiological response to specific challenges. We subjected females to a standardized restraint stress protocol [78], allowing us to measure the CORT response to a stressor via activation of the HPA axis. We also performed a GnRH challenge, testing the sensitivity of the HPG axis [79]. Finally, we analysed the effects of the social environment on female fecundity by measuring egg production and egg mass, fertilization rate, and number of offspring hatching in the F1 generation. To analyse potential differences between females within groups due to variation in affiliative and sexual interactions, we recorded social proximity and female baldness caused by repeated copulation with the male [72, 80].

We predicted group housing would result in elevated plasma androgen and CORT levels and higher yolk T levels due to the increased amount of social stimulation. We expected the change in circulating hormone levels to affect reproductive performance, however, given the variable results reported in the literature, we did not have a clear prediction regarding the direction of effects.

## Methods

### Experimental design

**Fig 1:**
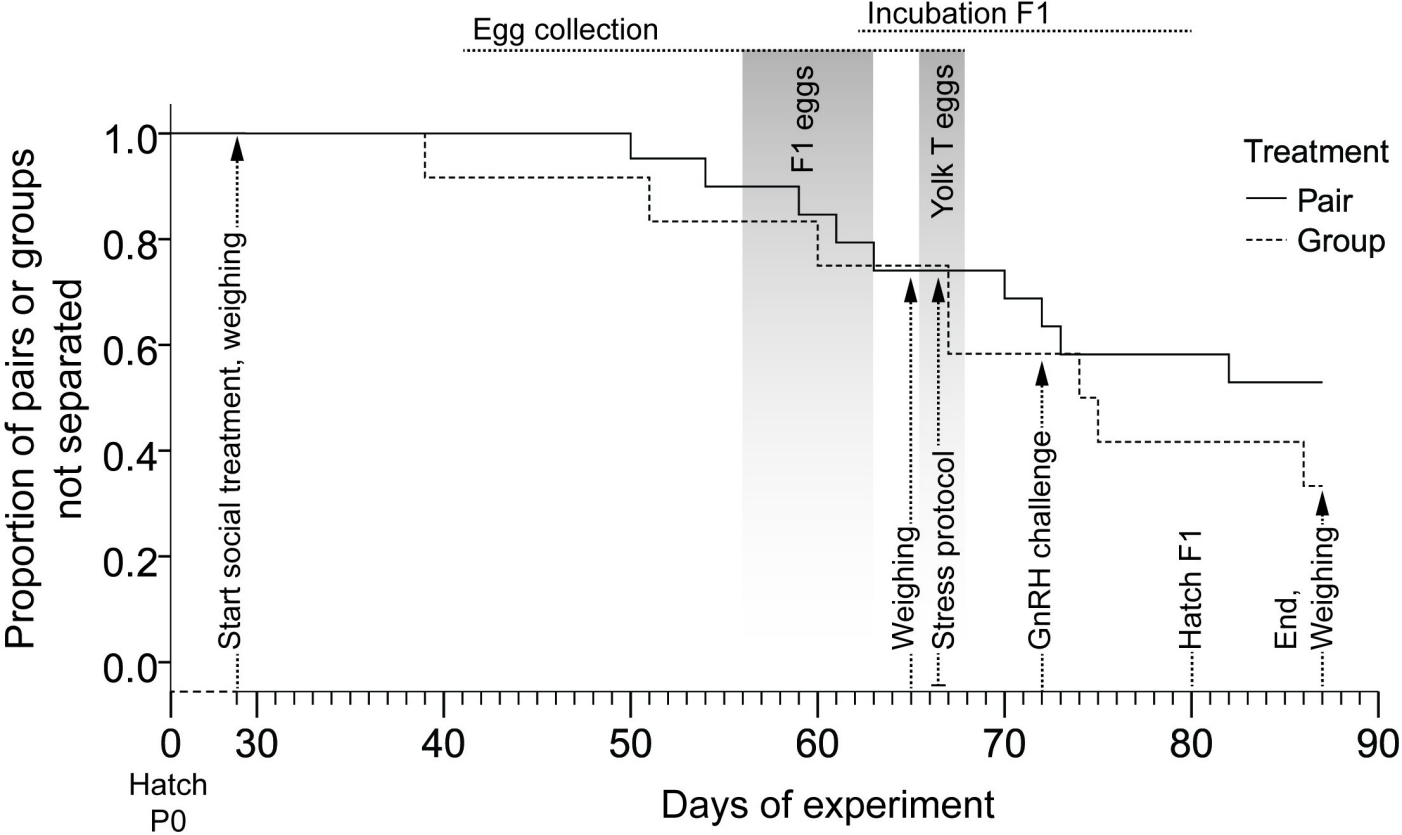
Timeline of experimental procedures, and separations of pairs and groups.

The experiment was conducted using a total of 96 animals. At 29 days of age, when the birds had developed their sexually dimorphic plumage but were not yet sexually mature, they were placed in the social treatment conditions. Groups consisted of three females and one male, while pairs included one female and one male. The birds were allocated as follows: 36 females and 12 males to 12 groups, 24 females and 24 males to 24 pairs. Siblings and half-siblings were equally distributed over the two social treatments and never housed in the same cage, in order to balance out potential genetic effects on endocrinology and reproduction. The distribution of the cages within the two experimental rooms was balanced for treatment. Measures of the females’ physiology, behaviour and reproduction were taken at different time points as described below (for an overview see Fig 1). Animals were weighed at the start of treatment (day 29), after five weeks into the treatment (day 65) and at the end of the experiment (day 87).

Due to aggression, we had to separate three pairs using a wire mesh which still allowed acoustic, visual, olfactory and limited tactile interaction. Two groups also had to temporarily be wire separated due to aggression (one animal on one side of the wire, the other three animals on the other side). Wire separated animals were included in our analyses and excluding these animals from our statistical analyses did not qualitatively change the results. Eight groups–including the two groups that had already been wire separated—and eleven pairs had to be completely removed from the experiment between day 30 and day 87 because at least one bird in the cage had wounds that were unlikely to heal within a few days, constituting a humane endpoint. In addition, in two pairs the male died, hence the females were excluded from all analysis of parameters following the death. As a consequence, sample size varies for different measures (Table 1). However, the reduction of sample size over the course of the experiment did not differ between social treatments (Kaplan-Meier survival analysis using Breslow test statistics: χ^2^_1_ = 1.06, p = 0.30; Fig 1).

**Table 1:** Measures of pair-housed and group-housed females and their offspring, with sample sizes.

Measure		Pair-housed females				Group-housed females			
		Mean ± 1 SEM	n	Excluded	Missing sample	Mean ± 1 SEM	n	Excluded	Missing sample
Female weight, day 65		235.09 g	17 ♀♀	7 ♀♀		230.10 g	33 ♀♀	3 ♀♀	
		± 5.35				± 2.60	11 groups	1 group	
Female weight, day 87		243.85 g	13 ♀♀	11 ♀♀		241.67 g	12 ♀♀	24 ♀♀	
		± 6.64				± 6.41	4 groups	8 groups	
Age at first egg		45.48 days	23 ♀♀	1 ♀		45.47 days	36 ♀♀		
		± 0.74				± 0.57	12 groups		
Total nr of eggs collected		0.66 eggs/♀/day	361 eggs	1 ♀		0.60 eggs/♀/day	533 eggs		
		± 0.02	23 ♀♀			± 0.02	12 groups		
Egg mass		10.17 g	324 eggs	1 ♀		10.33 g	531 eggs		
		± 0.06	23 ♀♀			± 0.04	12 groups		
Stress protocol	Baseline	2.77 ng/ml	14 ♀♀	6 ♀♀	4 ♀♀	2.12 ng/ml	24 ♀♀	3 ♀♀	9 ♀♀
		± 0.22				± 0.14	11 groups		
	Post-challenge	13.05 ng/ml	14 ♀♀			11.05 ng/ml	22 ♀♀	1 group	11 ♀♀
		± 6.20				± 7.74	11 groups		
Yolk mass		2.78 g	51 eggs	6 ♀♀		2.78 g	73 eggs	6 ♀♀	
		± 0.04	18 ♀♀			± 0.03	10 groups	2 groups	
Yolk T	Concentration	5.02 pg/mg	16 eggs	6 ♀♀	2 ♀	5.81 pg/mg	23 eggs	6 ♀♀	7 ♀♀
		± 0.89				± 0.86			
	Total	14.07 ng	16 ♀♀			15.86 ng	9 groups	2 groups	
		± 2.55				± 2.29			
GnRH challenge	Baseline	0.67 ng/ml	16 ♀♀	7 ♀♀	1 ♀	0.56 ng/ml	22 ♀♀	12 ♀♀	2 ♀♀
		± 0.05				± 0.03	8 groups		
	Post-challenge	0.72 ng/ml	16 ♀♀			0.72 ng/ml	22 ♀♀	4 groups	
		± 0.05				± 0.05	8 groups		
Eggs for F1	Eggs collected	6.89 eggs/♀	124 eggs	5 ♀♀	1 ♀^1^	6.46 eggs/♀	155 eggs	6 ♀♀	6 ♀♀^1^
		± 0.31				± 0.44			
	Eggs fertilized	6.06 eggs/♀				5.08 eggs/♀			
		± 0.30	18 ♀♀			± 0.55	8 groups	2 groups	
	Eggs hatched	3.72 eggs/♀				2.88 eggs/♀			
		± 0.46				± 0.52			
F1 offspring	Mass at hatching	7.18 g	66 chicks	5 ♀♀	2 ♀♀^1^^,^ ^2^	7.10 g	69 chicks	6 ♀♀	10 ♀♀^1^^,^ ^2^
		± 0.08				± 0.06			
	Tarsus at hatching	12.93 mm	17 ♀♀			12.77 mm	20 ♀♀	2 groups	
		± 0.06				± 0.07	8 groups		

^1^male infertile

^2^females without offspring.

### Animal husbandry

The Japanese quail originated from eggs generously provided by the INRA in Nouzilly, France (Experimental unit 1295 (UE PEAT) and UMR 85, Physiologie de la Reproduction et des Comportements, INRA-CNRS-IFCE-Université de Tours, Val de Loire Center, Nouzilly, France). Eggs were laid by females from a non-selected control line, bred next to quail lines selected for low or high social reinstatement [81]. At the INRA, each cage housed two females and one male, thus housing conditions were intermediate compared to the conditions used in this study. All eggs were incubated at the same time, hatched, and birds reared at Bielefeld University, Germany.

The experiments were performed in two adjacent indoor rooms with artificial lighting and no natural daylight. The light-dark cycle was 14:10 h (lights on at 5:00 am, lights off at 7:00 pm), and the rooms had ambient temperature with additional heating to maintain at least 20°C. Cages for pairs measured 75 x 80 x 40 cm, group cages 150 x 80 x 40 cm. None of the cages faced each other to prevent visual contact between birds from different cages, but acoustic and olfactory communication was possible. The birds were kept on wood shavings, and all cages contained a sand bath and one shelter hut per female. Feed (GoldDott Hennenmehl, Derby Spezialfutter GmbH, Münster, Germany) and water was provided ad lib. The standard diet was supplemented on a weekly basis with mealworms and shell grit.

### Ethics statement

All experimental procedures and humane endpoints for minimizing suffering were approved by the North Rhine-Westphalia State Agency for Nature, Environment and Consumer Protection (Landesamt für Natur, Umwelt und Verbraucherschutz Nordrhein-Westfalen), Recklinghausen, Germany (licence number 84–02.04.2013-A127). Animal facilities were approved for keeping and breeding Japanese quail for research purposes by the local government authority responsible for health, veterinary and food monitoring (Gesundheits-, Veterinär- und Lebensmittelüberwachungsamt Bielefeld, Germany).

### Egg collection

All cages were checked for eggs daily from day 39 after hatching, 10 days after birds had been placed in the experimental groups and before any egg had been laid. Eggs were collected until day 68 and all eggs were weighed to the nearest 0.01 g. Eggs collected until day 56 were used to analyse the onset of egg laying in pair-housed vs. group-housed females. Since we could not identify which female laid an egg in a group, we recorded the day at which we found the first, second and third egg in a group as the age at which the first, second and third female had started laying eggs. Eggs collected between day 56 and day 63 were artificially incubated to produce the F1 generation (see Table 1 for sample sizes). Eggs from days 66–68 were frozen at -20 directly after collection and later used to determine yolk T concentrations (see Table 1 for sample sizes). Most eggs for yolk T measurements were collected in the morning before the stress protocol (n = 31), or the morning of the day after the stress protocol (n = 7). This means that at the time of the stress protocol, most eggs for yolk T measurements had already been laid or ovulated, and yolk T levels were unlikely to be affected by the stress protocol (in quail, ovulation usually takes place in the afternoon, approximately 15–30 minutes after oviposition of the previous egg [82–84]). Only one egg was collected in the morning two days after the stress protocol, but here too the yolk should have been almost completely formed at the time of the stress protocol. Since deposition of yolk T is repeatable within individual females [85], we expected the yolk T measurements to be representative for all eggs that an individual female laid during the course of this study. All females continued laying until the end of the experiment (day 87). In total, we collected 361 eggs from pair females and 533 eggs from group females (see Table 1).

### Egg incubation and hatching

Eggs were incubated in complete darkness in a HEKA-Euro-Lux II incubator (HEKA-Brutgeräte, Rietberg, Germany). Until incubation day 14, the temperature was set at 37.8°C, humidity to 55% and eggs were turned for 30 minutes every 2 hours. Candling of the eggs was done after 9 days of incubation, and infertile eggs were removed. On day 15 of incubation, eggs were moved to hatching trays, the incubation temperature was set to 37.5°C, the humidity to 75%, and eggs were no longer turned. On average, chicks hatched after 17 days of incubation (range: 16–18 days). Chicks were removed from the incubator once their feathers had dried (ca. 2 hours after hatching). Upon removal from the incubator, all chicks were weighed to the nearest 0.1 g and tarsus was measured to the nearest mm using a digital calliper. Parentage for offspring of group-housed mothers was ascertained by genotyping all parents and chicks. A small blood sample (max. 50 μl) was taken from the chicks on the day of hatching by pricking the jugular vein with a 27 gauge needle and collecting the blood in heparinized capillaries (BRAND GMBH + CO KG, Wertheim, Germany). From the parental birds, a small sample of blood from the stress protocol or GnRH challenge were used. Blood was diluted 1:2 with phosphate buffer saline (10 mM PBS+6 mM EDTA, pH 7.4) and stored at -20°Celsius.

For genotyping, genomic DNA was obtained by a phenol/chloroform or Chelex extraction [86]. We genotyped all individuals at 22 microsatellite loci using fluorescently labelled primers selected from a previous study [87] in three separate multiplexed PCR reactions: mix 1 (GUJ0001, GUJ0011, GUJ0028, GUJ0044, GUJ0068, GUJ0085, GUJ0097, GUJ0100; annealing temperature 55° Celsius), mix 2 (GUJ0021, GUJ0029, GUJ0062, GUJ0065, GUJ0069, GUJ0074, GUJ0083, GUJ0094; annealing temperature 60° Celsius), mix 3 (GUJ006, GUJ0054, GUJ0057, GUJ0071, GUJ0077, GUJ0092; annealing temperature 55°Celsius). DNA was amplified using the Type-It Kit (Qiagen) in 10 μl reactions (1 μl DNA, 1 μl primer-mix, 3.5 μl Type-It mastermix, 4.5 μl water), following the manufacturer’s PCR protocol (one cycle of 5 min at 94°C; 28 cycles of 30 s at 94°C, 90 s at the annealing temperature and 30 s at 72°C; and one final cycle of 15 min at 72°C). PCR products were separated by electrophoresis on a capillary sequencer (ABI 3730xl), fragment sizes were scored automatically using GeneMarker v1.95 and checked manually for errors. Paternity was manually assigned by identifying which genotype of the three potential mothers in a cage matched the offspring genotype.

### Stress protocol

Female CORT baseline and response values after a stressor were assessed in a standardised restraint stress protocol on days 66–67. All birds were tested between 09:15 am—12:30 pm and CORT levels did not change significantly during that period (F_(1, 30.89) time of day_ = 2.83, p = 0.10). Birds were caught, and a blood sample was taken within 3 minutes to determine baseline plasma CORT concentrations. Blood sampling was done by puncturing the ulnar vein with a sterile needle and collecting 200–300 μl blood in heparinised capillaries (BRAND GMBH + CO KG, Wertheim, Germany). Following the baseline sample, the birds were restrained for 10 minutes by placing them in bird holding bags (Ecotone, 25x30 cm), after which a second blood sample was taken to determine the CORT response to restraint.

### GnRH challenge

On day 72 we measured the females’ baseline plasma androgen concentrations, and their response values following a GnRH injection while females were still laying eggs. To exclude effects of the GnRH injection itself on yolk hormone levels or reproduction, the GnRH challenge was performed after collecting the eggs for the next generation and for yolk T measurements [68]. All birds were tested between 10:00 am—15:30 pm. As in the stress protocol, animals were caught, and a blood sample was taken from the ulnar vein within 3 minutes to determine baseline plasma androgen concentrations. After the baseline sample was taken, the females were injected in the pectoral muscle with 5 μg chicken GnRH-I (H-3106, APC number 54-8-23, CAS No: 47922-48-5, Bachem, Bubendorf, Switzerland, formerly also sold as Sigma-L0637) dissolved in 50 μl PBS, based on a protocol previously used in quail [68], and returned to their home cages. Thirty minutes post injection, the birds were caught again, and a second blood sample was taken to determine the androgen response to GnRH.

### Social proximity and baldness scores

To assess social proximity, all cages were checked once a day in the morning from day 45 to day 63 (except for weekends, resulting in 16 daily checks) to note which individuals were sitting together (within the space of one quail body length from each other). We then calculated how often a female sat with the male and, in groups, how often a female sat with at least one other female.

As a measure of male copulatory behaviour with the female, we classified females as ‘bald’ or ‘not bald’ depending on whether feathers were missing from the back of their head or their back at the end of the experiment (on day 87 or on the day of separation for separated animals). Male Japanese quail grab the female’s head or neck feathers during copulation and then mount her back [72,80]. Hence, baldness occurs following frequent copulations. We did not score baldness on a location other than the back of the head or the back involving skin damage since this is most likely caused by aggressive pecking.

### Plasma corticosterone and androgens

Blood samples from both the stress protocol and the GnRH challenge were kept on ice for a maximum of two hours after sampling and then centrifuged for 10 minutes at 2000 x g. Following centrifugation, plasma was collected and frozen at -20°C.

Plasma CORT concentrations were determined using a commercial corticosterone radioimmunoassay kit (MP Biomedicals, Orangeburg, USA, cat. no. 07–102102). Cross-reactivity of the kit antibody, as reported by the manufacturer, was 0.34% for desoxycorticosterone, 0.1% for testosterone, and less than 0.1% for other steroids. Samples were balanced for treatment across assays. Samples were measured together with quail plasma samples from other experiments and all were distributed over 10 assays with an average intra-assay coefficient of variation (CV) of 4.78%, and an inter-assay CV of 7.13% (based on a chicken plasma pool and 2 kit controls measured in each assay).

Plasma androgen concentrations were determined using a commercial T enzyme immunoassay kit (Demeditec Diagnostics GmbH, Kiel, Germany, cat. no. DES6622). Cross-reactivity of the kit antibody, as reported by the manufacturer, was 23.3% for 5α-dihydrotestosterone, 1.6% for androstenedione, and less than 0.1% for other steroids. Control plasma pool samples were incorporated in each run. Samples from the third assay were significantly higher than samples measured in the first two assays (effect of assay: F_(2, 18.01)_ = 5.93, p = 0.01). Since eight samples (four each from assay one and two) were re-measured in assay three, we could correct the values from the third assay using a regression of the measures for samples re-measured in assay three on their corresponding values from assays one and two. Excluding samples from assay three did not change the results qualitatively. After correction, the average intra-assay CV was 5.84% and the inter-assay CV was 9.77%.

### Yolk testosterone

Yolk preparation and extraction was based on previously established methods [88]. In preparation for extraction, the frozen yolk was separated from the albumen and egg shell and weighed to the nearest 0.01 g. Yolks were homogenized with 4 ml distilled water and then stored at -20°C.

For T extraction, 100 mg of the yolk-water mix was transferred to a 2 ml Eppendorf tube and further diluted with 100 μl distilled water. All samples were then spiked with 4000 cpm of tritium-labelled T (Perkin Elmer NET553250UC), vortexed and incubated for 30 minutes in a 37°C water bath. After incubation, 500 μl 100% ethanol was added, and samples were vortexed for 15 minutes. After vortexing, samples were centrifuged for 10 minutes at 4°C and 15800 x g. The supernatant was then decanted into a fresh 2 ml Eppendorf tube and frozen overnight on dry ice. The next day, samples were centrifuged again for 10 minutes at -9°C and 15800 x g, and the supernatant was decanted into fresh tubes. Samples were then dried in a vacuum concentrator (approximately 2 hours) and the pellet was re-dissolved in 500 μl steroid-free human serum (IBL international, RE52999). For determination of extraction efficiency, 30 μl of each sample was counted in a beta counter. Recoveries were on average 82.5 ± 0.8% (mean ± 1 SEM).

A commercial radioimmunoassay kit was used to determine yolk T concentrations (DIAsource ImmunoAssays S.A., Louvain-la-Neuve, Belgium, cat. no. KIP1709). Cross-reactivity of the kit antibody, as reported by the manufacturer, was 0.31% for dihydrotestosterone, 0.28% for androstenedione and less than 0.1% for other steroids. All samples were measured in a single assay, the intra-assay CV was 3.86%. Total yolk T was calculated by multiplying the yolk T concentrations with total yolk mass.

### Statistical analyses

Statistical analyses were performed using R 3.2.3 (R Development Core Team 2015). General linear mixed models were fitted for plasma hormones, yolk T, growth, onset of egg laying, egg mass, yolk mass and F1 mass and tarsus at hatching (calculated using lmer from the lme4 package in R, using the package lmertest to extract F values and p values). Pearson’s correlation coefficient was used to analyse the correlation between baseline and post-challenge androgens and CORT. Generalised linear mixed models (calculated using glmer from R’s lme4 package) with a binomial distribution and logit link function were calculated for analysis of egg laying, fertilization and hatching success.

For the analysis of hormonal responses to the GnRH challenge and the stress protocol, we used cage and individual identity (ID) nested within cage as random effects and social treatment (treatment) and sample number (sample) as fixed predictors, as well as the treatment by sample interaction (treatment * sample).

To analyse potential effects of copulation with the male, or social proximity on female baseline plasma hormone concentrations, we fitted models on baseline androgen and CORT concentrations, with female baldness (baldness; since there were only three non-bald pair-housed females, they were excluded from the analysis and we compared three categories: bald pair-housed females, and bald and non-bald group-housed females) or social proximity (sitting with male or sitting with female) as fixed predictors. Least-significant-difference post-hoc tests were used to test which categories differed from each other. All models included cage as a random effect.

Yolk T was analysed using cage as a random effect and treatment as a fixed effect, and to test the relationship between plasma androgens and yolk T, the models included either average baseline androgen concentration (baseline androgens) or average plasma androgen increase in response to GnRH (Δ androgens in response to GnRH) from each cage as an additional predictor. The average female plasma androgen concentration per cage was used since we were unable to assign eggs to individual females in groups.

Analyses of female mass included cage and individual ID nested within cage as random effects, treatment and female age (age) as fixed predictors, and female mass at the start of the social treatment (day 29) as a covariate.

Models analysing the onset of egg laying (age at first egg) included a random effect of cage, and social treatment as a fixed predictor.

Models for egg laying rate and egg mass included a random effect of cage and a fixed effect of social treatment. In addition, the models analysing egg laying rates included a linear, quadratic and cubic effect of collection day (collection day + collection day^2^ + collection day^3^) to model the non-linear relationship between age and egg laying rates. Likewise, the models for the analysis of egg mass included a linear, quadratic and cubic effect of the days since the onset of laying for each cage (day+day^2^+day^3^). Moreover, egg laying rate models included a treatment by age interaction effect (treatment *(collection day + collection day^2^ + collection day^3^)), and egg mass models an effect of the interaction of treatment and days since the onset of egg laying (treatment * (day+day ^2^+day^3^)).

Yolk mass was only measured in the subset of eggs collected for yolk hormone measurements, and models for the analysis of yolk mass included a random effect of cage and a fixed effect of social treatment.

Fertilization and hatching success were analysed using cage as a random effect and treatment as a fixed effect.

F1 mass and tarsus at hatching included maternal cage and maternal ID nested within maternal cage as random effects (we were able to include maternal ID since we had assigned chick parentage) and treatment as a fixed effect.

We started out with the full models including all interactions and then excluded stepwise all non-significant predictors/interactions (p > 0.05), except for the main terms of interest, i.e. social treatment and sample number (for hormonal responses). Distributions of model residuals were tested using Kolmogorov-Smirnoff tests and visually assessed using histograms and Q-Q plots. Plasma CORT concentrations were log-transformed, and yolk T concentrations were square-root transformed to achieve normality. No transformation was used when analysing the correlation between baseline plasma androgens and CORT.

## Results

### Androgens

**Fig 2:**
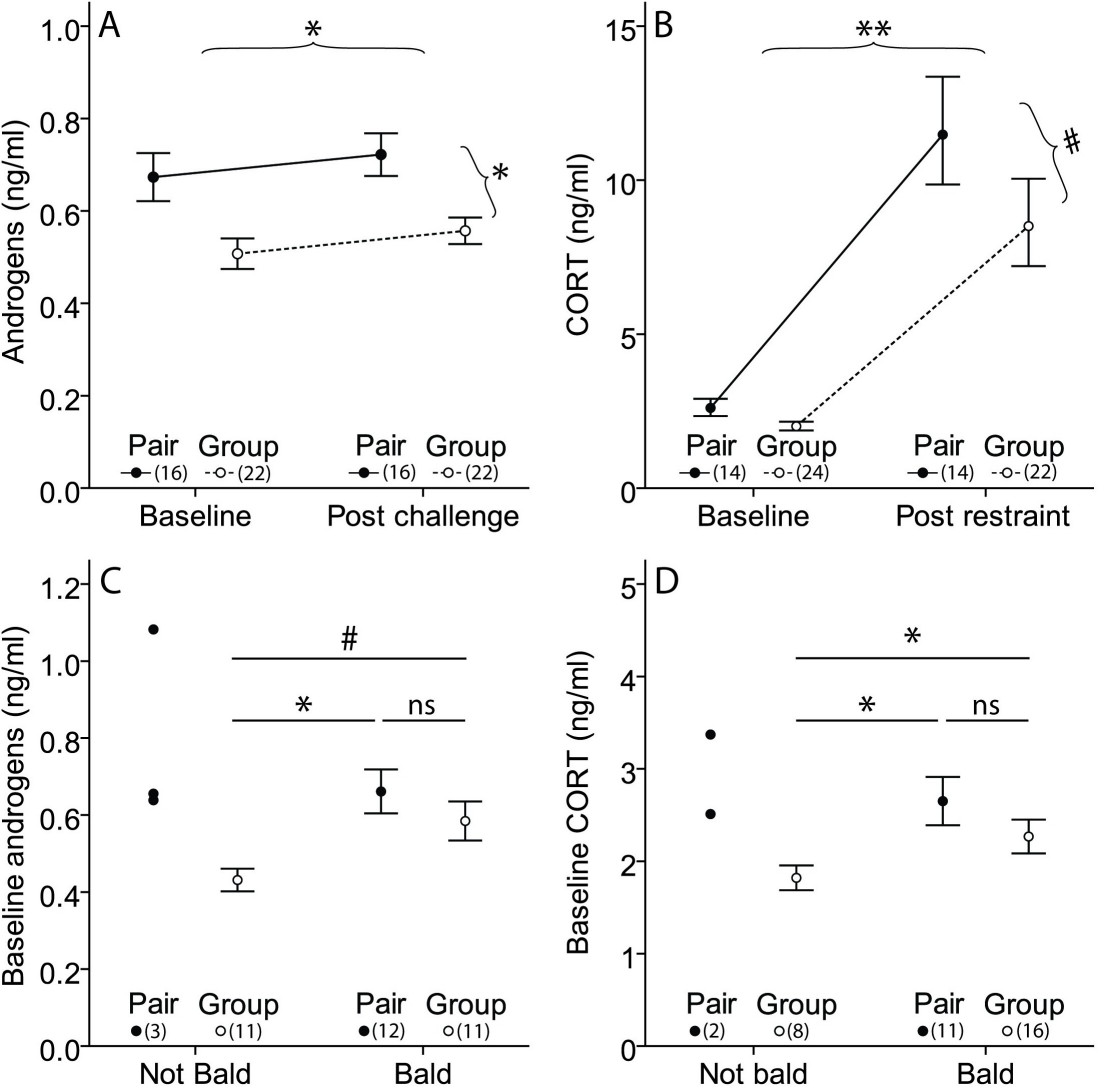
Plasma hormones. (A) plasma androgen concentration in ng/ml before and 30 minutes after an injection with 5 μg GnRH. (B) plasma CORT concentration in ng/ml before and after being restrained for 10 minutes (backtransformed from Log10). (C) baseline plasma androgen concentration and (D) baseline plasma CORT concentrations in ng/ml of bald and non-bald pair-housed females, and bald and non-bald group-housed females. Data from bald pair-housed females are indicated by solid circles, but were not used in the statistical analyses. Data are shown as means ± 1 SEM. Numbers between brackets indicate sample sizes. ** = p < 0.01; * = p < 0.05; # = p < 0.1; ns = not significant.

Overall, pair-housed females had significantly higher plasma androgen concentrations than group-housed females (F_(1, 11.40) Treatment_ = 7.88, p = 0.02; Table 1, Fig 2A). GnRH injections resulted in a small, but significant, increase in plasma androgen concentrations (F_(1, 37) Sample_ = 6.46, p = 0.02; Table 1, Fig 2A), but the response to GnRH did not differ between pair-housed and group-housed females (F_(1, 36) Treatment * sample_ < 0.01, p = 0.98; Fig 2A).

### Corticosterone

Overall, pair-housed females had slightly higher plasma CORT concentrations than group-housed females, but the difference did not reach statistical significance (F_(1, 28.13) Treatment_ = 3.61, p = 0.07; Table 1, Fig 2B). 10 minute restraint significantly increased plasma CORT concentrations (F_(1, 53.93) Sample_ = 137.06, p < 0.01; Table 1, Fig 2B), but the increase did not differ between social treatments (F_(1, 52.90) Treatment * sample_ = 0.02, p = 0.88; Fig 2B). Individual baseline plasma CORT concentrations were not significantly correlated with baseline plasma androgen concentrations (r_(28)_ = 0.23, p = 0.22; S1A Fig) and post-challenge plasma CORT concentrations did not correlate with post-challenge androgen levels (r_(27)_ = 0.21, p = 0.26; S1B Fig).

### Social proximity, baldness and baseline plasma hormones

In both pairs and groups, the proportion of time a female spent sitting with the male did not predict baseline plasma androgen or CORT concentrations, nor did the proportion of time a female spent sitting with at least one other female in her group (All F-values < 1.32, all corresponding p-values > 0.26; S2A–S2D Fig).

Bald pair-housed females, bald group-housed females and non-bald group-housed females differed significantly in their baseline plasma androgen and CORT concentrations (baseline androgens: F_(2, 6.89) baldness_ = 5.24, p = 0.04; Fig 2C baseline CORT: F_(2, 28.55) baldness_ = 3.48, p = 0.045; Fig 2D). Non-bald group-housed females had significantly lower baseline plasma androgen concentrations than bald pair-housed females and had marginally lower baseline androgen concentrations than bald group-housed females, although the latter was not statistically significant (t_(7.17)_ = 3.21, p = 0.02 and t_(5.64)_ = 2.12, p = 0.08 respectively). In addition, non-bald group-housed females had significantly lower baseline plasma CORT concentrations than both bald pair-housed females and bald group-housed females (t_(26.26)_ = 2.52, p = 0.02 and t_(29.13)_ = 2.07, p = 0.048 respectively). Baseline plasma androgen and CORT concentrations did not differ between bald pair-housed and bald group-housed females (baseline androgens: t_(9.07)_ = 1.00, p = 0.35; baseline CORT: t_(21.04)_ = 0.85, p = 0.40). Since there were only three non-bald pair-housed females that were still together with their male at the time of the hormone measurements, we did not include them in the statistical analysis. However, their androgen (n = 3) and CORT (n = 2) values were higher than most non-bald group-housed females and as high as most of the bald females in both social treatments (Fig 2C and 2D).

### Yolk testosterone

We averaged yolk T values for each cage because eggs could not be assigned to individuals for group-housed females. Despite the treatment differences in plasma androgen concentrations, yolk T concentrations did not differ between the social treatments (F_(1, 21.49) Treatment_ = 0.33, p = 0.57; Table 1), nor did total yolk T levels (F_(1, 22.99) Treatment_ = 0.22, p = 0.64; Table 1). Neither average baseline plasma androgen concentrations nor the average response to GnRH predicted yolk T concentrations (F_(1, 18.00) Baseline T_ = 0.28, p = 0.60; F_(1, 18.00) ΔT in response to GnRH_ = 1.23, p = 0.28; S3A and S3B Fig). We could directly correlate female plasma androgen and yolk T concentrations only in pair-housed females, but analysis also showed no relationship between the two (F_(1, 13) Baseline androgens_ = 0.01, p = 0.92; F_(1, 13) Δ androgens in response to GnRH_ = 0.42, p = 0.53; S3A and S3B Fig).

### Growth and reproductive performance

**Fig 3:**
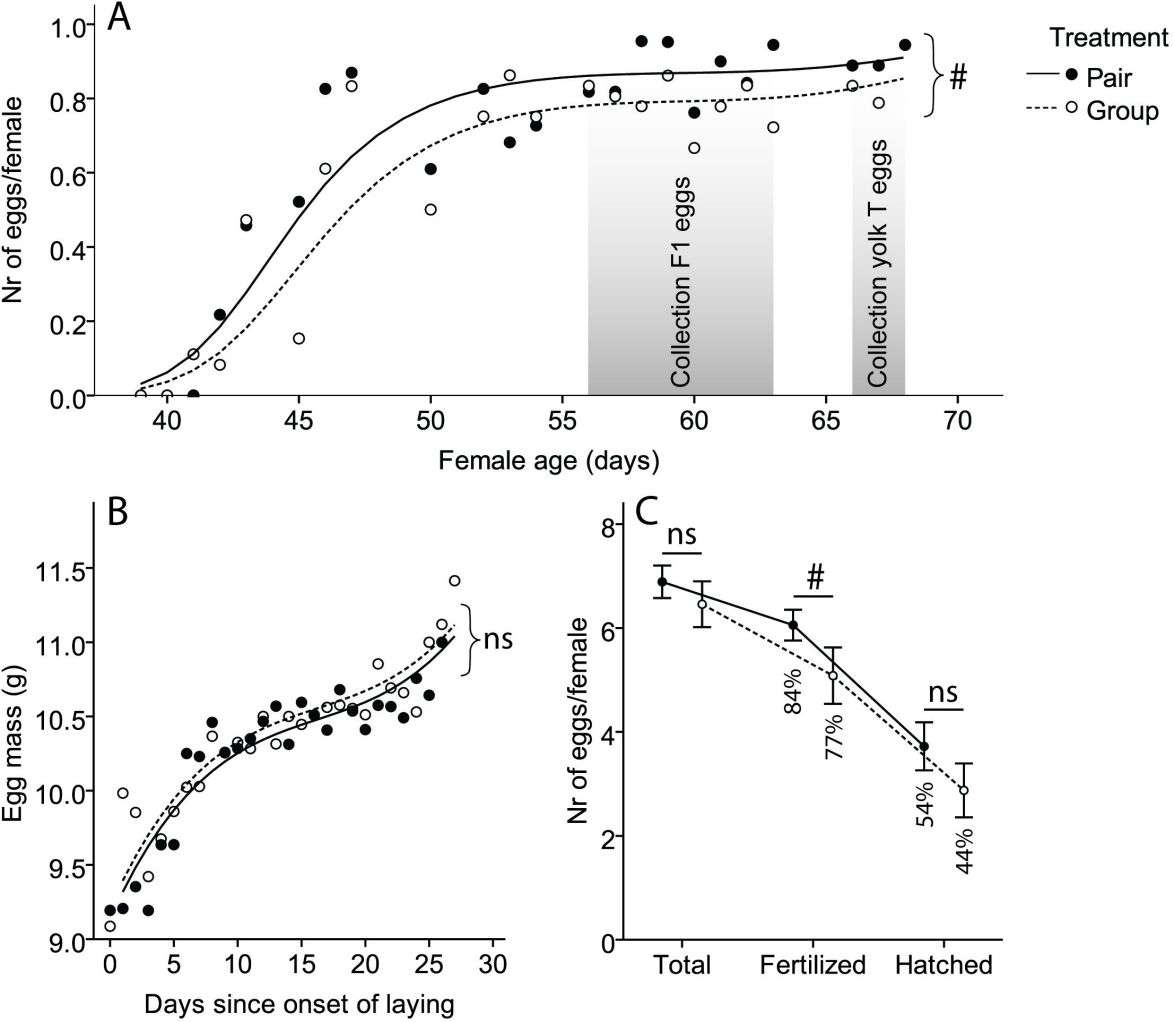
Measures of reproductive performance. (A) Egg production for both social treatments. Circles show the mean number of eggs/female found on a given day. Lines show the model predictions. n = 361 eggs from 23 pairs and 533 eggs from 12 groups. (B) Egg mass for both social treatments. Circles show the mean egg mass, lines the model predictions. n = 324 eggs from 23 pairs and 531 eggs from 12 groups. (C) Average number of eggs collected for F1 and the number and percentage of eggs that were fertilized and hatched, per social treatment. n = 124 eggs from 18 pairs, 155 eggs from 8 groups. Error bars indicate ± 1 SEM. * = p < 0.05; # = p < 0.1; ns = not significant.

Female growth was not affected by the social environment (F_(1, 31.16) Age * treatment_ = 1.05, p = 0.31; Table 1, S4 Fig). The first eggs were laid on day 41, and by day 58 all females were laying, but the onset of egg laying did not differ between the social treatments (F_(1, 28.27) Treatment_ < 0.01, p > 0.99; Table 1). Egg laying rates were higher in pair-housed females compared to group-housed females, but not significantly so (treatment: χ^2^_(1)_ = 3.62, p = 0.06, Fig 3A). For all females, the number of eggs laid increased over time ((collection day + collection day^2^ + collection day^3^): χ^2^_(3)_ = 529.30, p < 0.01, Fig 3A), and there was no significant difference in this increase between the social treatments (treatment*(collection day + collection day^2^ + collection day^3^): χ^2^_(3)_ = 4.57, p = 0.21; Fig 3A).

Egg mass was not affected by social treatment (treatment: χ^2^_(1)_ = 0.17, p = 0.68; Table 1, Fig 3B), but it increased significantly over time ((day+day^2^+day^3^): χ^2^_(3)_ = 328.50, p < 0.01; Fig 3B). The increase in egg mass did not differ between treatments (treatment*(day+day^2^+day^3^): χ^2^_(3)_ = 3.04, p = 0.39; Fig 3B). Treatment did not affect yolk mass in the subset of eggs collected for yolk hormone analysis (F_(1, 21.82) Treatment_ = 0.13, p = 0.72; Table 1).

Pair-housed females on average laid almost one more fertilized egg per female than group housed females, but the difference in fertility did not reach statistical significance (z = -1.72, p = 0.09; Table 1, Fig 3C). Hatching success (the percentage of all eggs collected for the F1 generation that hatched, i.e. including infertile eggs) did not differ between treatments (z = -1.16, p = 0.25; Table 1, Fig 3C). Hatchlings did not differ in body mass (F_(1, 11.45) Treatment_ = 0.02, p = 0.91; Table 1) or tarsus length (F_(1, 16.02) Treatment_ = 0.36, p = 0.56; Table 1).

## Discussion

In many vertebrates, the social environment affects physiology and behaviour with consequences for female reproductive performance and offspring quality, but the underlying mechanisms are not well understood. We therefore investigated how the social environment—pair or group housing—of Japanese quail females affects their reproductive and stress physiology, yolk hormone deposition, and fecundity. Contrary to our expectations, female quail housed in pairs had higher plasma androgen concentrations and slightly higher CORT concentrations than females housed in groups, although the latter did not reach statistical significance. Treatment did not affect the HPG-axis or HPA-axis response to standardized challenges, nor were there differences in yolk T levels or fecundity. Female body mass, hatchling numbers, weight, and size were not affected by the social environment. Because baseline CORT levels were similar to plasma CORT concentrations found before in quail [36, 89–91] and females responded to the stress protocol with a significant increase in CORT, it is unlikely that our birds had experienced chronic stress, potentially masking any treatment effects.

Previous studies found increased plasma androgen and CORT concentrations in females exposed to increased social stimulation, higher social density or social instability [16, 17, 35, 92, 93]. We suggest that we found the opposite because of differences in the adult sex ratio between the social treatments, leading to differences in male-female interactions. In the present study, the males’ attention in group cages was divided among three different females, whereas in pairs there was only one female to interact with. As a consequence, the effect of the male’s presence on circulating female androgen and CORT concentrations was likely to be stronger in pairs than in groups. Indeed, previous studies have shown that the sex composition of groups [94], male courtship song [95] and copulation with a male [91] can affect a female’s endocrine status, including androgen and CORT levels. Higher male:female ratios induced stress in groups of domestic chicken [94], and male courtship song increased female circulating androgen levels in canaries [95]. In Japanese quail, copulation with a male increased female CORT levels, while the number of mounts by the male and male body condition positively correlated with the female’s androgen response to copulation [91]. Japanese quail males often engage in forced copulations, which has been suggested as a source of stress for females [36, 74, 96, 97]. Interestingly, in the group treatment, bald females, who presumably experienced more copulation attempts by the male, had higher baseline androgen and CORT levels than non-bald females and levels that were more similar to those of pair-housed females. In addition, there were hardly any non-bald females in pairs. This supports the idea that the intensity of male-female interactions is an important factor affecting female circulating hormone concentrations. However, it remains to be investigated whether the results found in the present study were indeed due to an effect of copulation with the male or due to other aspects of the treatment. In addition, the social environment most likely affects male endocrine status as well, which may have important consequences for male-female interactions [98–100] and should be taken into account in future studies.

Effects of the adult sex ratio may not be solely due to an influence of males, but also due to the number of females present. Female Japanese quail prefer to associate with other females when a male is present, indicating that females may try to avoid unwanted sexual attention by the male by grouping together [101]. In the present study, the effects of the male may therefore be alleviated in groups due to social buffering, although we did not find a correlation between female-female social proximity and baseline hormone levels.

Although we did not find an effect of social proximity on hormone levels, we cannot rule out potential effects of cage size. Since pairs were housed in cages that were 50% smaller than cages for groups to ensure a comparable social density between treatments, the differences in cage size may have affected individual movement patterns and use of space [102] and thereby social interactions and social avoidance behaviour in particular.

Several studies in birds have shown that breeding density, social instability, social motivation and female-female competition is positively correlated with yolk androgen concentrations, yolk T in particular [17, 35, 57–62, 81, 103]. In contrast, we did not find effects of group size on average yolk T in the present study. Social instability did lead to increased yolk androgen levels in a previous study on Japanese quail [35]. We expected a similar effect in group-housed females because we assumed that group living would result in a less stable social environment than pair housing since females would encounter and interact with multiple individuals. However, groups may have been less stable than pairs only at the start of the social treatments when group-housed individuals had to familiarize themselves with more conspecifics than pair-housed birds. Since the social environment remained stable over time in both treatments and eggs for yolk T measurements were collected 37 days after the start of the social treatments, the females likely had ample time to familiarize with their group members, and any initial differences in yolk T levels might have disappeared over the course of the experiment. Finally, variation between females within groups may have masked treatment differences in yolk T concentrations, which should be analysed in future studies as we unfortunately could not assign eggs to individual females in groups.

The magnitude of the plasma androgen response to GnRH has been proposed to be a better predictor of yolk androgen deposition than baseline plasma androgen concentrations [66, 67]. However, this has only been investigated by correlating a female’s androgen response to GnRH with yolk androgen levels measured in subsequently laid eggs [66–68]. In the present study, we assessed the female’s androgen response to GnRH during egg laying, but after we had collected eggs for yolk hormone measurement. Neither baseline plasma androgens nor the androgen response to GnRH predicted yolk T levels, suggesting that yolk T does not reflect the female’s inherent sensitivity to GnRH, but rather that stimulation by GnRH may affect yolk T deposition in eggs laid later. Another possibility is that the link between the GnRH response and yolk T may be dosage-dependent and context-dependent [66]. We found a significant increase in plasma androgen levels in response to GnRH injections, but the increase was smaller than that found in previous studies with Japanese quail using similar dosages of chicken GnRH-I [68]. This might be due to genetic or environmental differences between populations and studies, and a low response to GnRH may not be reflected in yolk T levels, as opposed to a high GnRH responsiveness.

Female reproductive performance was largely unaffected by the social treatments, indicating that the effects on female’s endocrine physiology had little fitness consequences. A possible explanation is that the differences in social treatments had little effect since domesticated Japanese quail have been heavily selected for egg production [70–74]. Moreover, since only a subset of eggs was incubated to calculate fertility and number of hatchlings, these differences might have been larger had all eggs been incubated. Although the difference did not reach statistical significance, it is noteworthy that pair-housed females had somewhat higher egg laying rates and fertility compared to group-housed females. Egg production and fertility may be slightly higher in pairs because the higher male:female ratio may have stimulated female reproduction [99, 104, 105], as demonstrated by the fact that most pair-housed females were bald and therefore experienced a higher rate of copulation. In addition, pair-housed quail might have had higher levels of within-pair testosterone covariation which has been found to positively predict reproductive output [106, 107] (but see [108]).

## Conclusion

Contrary to expectations, we found that increased group size did not result in elevated plasma androgen or CORT concentrations. Instead, we found higher circulating androgen and CORT levels in pair females, possibly due to a stimulating effect of a higher frequency and intensity of copulations with the male on female physiology. These treatment effects were not reflected in yolk T levels, and in contrast to previous studies the plasma androgen response to GnRH was not correlated with yolk T, suggesting independent regulation of plasma hormones and yolk hormones. In addition, there were no strong effects on reproductive performance. The unexpected finding of higher circulating androgen and CORT levels in pair-housed females demonstrate that we need a better understanding of how group sex ratios and specific aspects of male-female and female-female relationships and their interactions affect female endocrine physiology.

## Supporting information

S1 Fig Correlations between baseline and post challenge concentrations of androgens and CORT in female plasma. (A) Individual baseline plasma CORT concentrations plotted against baseline plasma androgen concentrations, and (B) individual post-challenge plasma CORT concentrations plotted against post-challenge androgen levels for both social treatments. Neither baseline nor post-challenge CORT and androgen concentrations were correlated with each other.
(TIF)

S2 Fig Social proximity and baseline plasma hormones. The proportion of all scan observations that a female was found sitting within a distance of one body-length from the male, plotted against the (A) baseline androgen concentration and the (B) baseline CORT concentration in her plasma, for both social treatments. The proportion of time a female spent sitting with the male did not predict baseline plasma CORT or androgen concentrations (CORT: F(1, 33.60) _Sitting with male_ = 0.02, p = 0.88; androgens: F(1, 33.45) _Sitting with male_ = 0.11, p = 0.75), and this was true for both social treatments (CORT: F(1, 32.95) _Treatment*sitting with male_ = 1.32, p = 0.26; androgens: F(1, 32.40) _Treatment*sitting with male_ = 0.45, p = 0.51). (C) and (D) show the proportion of scan observations that a group-housed female was found sitting close to another female, plotted against (C) her baseline plasma androgen concentration and (D) her baseline plasma CORT concentration. The proportion of time a female spent sitting with at least one other female did not predict baseline plasma CORT or androgen concentrations (CORT: F(1, 20.98) _Sitting with female_ = 0.26, p = 0.62; androgens: F(1, 17.07) _Sitting with female_ = 0.25, p = 0.63).
(TIF)

S3 Fig Correlations between plasma androgens and yolk T. (A) Average baseline plasma androgen concentrations per cage and (B) the average androgen response to GnRH per cage plotted against the average yolk T concentrations per cage. Note that in pair housed-females, the average concentrations are the same as the individual female’s concentrations as there is only one female per cage. For group-housed females, hormone values were averaged per cage because eggs could not be assigned to individuals within groups. Neither baseline plasma androgen concentrations, nor the response to GnRH predicted yolk T concentrations.
(TIF)

S4 Fig Female growth between day 65 and day 87. Depicted is the average body mass of both pair-housed and group-housed females, which did not differ between treatments, on day 19 (n = 24 pair-housed females and 36 group-housed females from 12 groups), 65 (n = 17 pair-housed females and 33 group-housed females from 11 groups) and day 87 (n = 13 pair-housed females and 12 group-housed females from 4 groups). Error bars indicate ± 1 SEM.
(TIF)

S1 Dataset Raw data. (XLSX)
