# Correction: Social environment during egg laying: Changes in plasma hormones with no consequences for yolk hormones or fecundity in female Japanese quail, *Coturnix japonica*

**DOI:** 10.1371/journal.pone.0199115

**Published:** 2018-06-11

**Authors:** Esther M. A. Langen, Nikolaus von Engelhardt, Vivian C. Goerlich-Jansson

There are errors in Table 1. Standard deviation is reported instead of standard error of the mean (SEM) for post-challenge stress protocol. Erroneous mean and SEM values are present for group-housed females in the GnRH challenge. Please see the corrected Table 1 here.

**Table 1:** Measures of pair-housed and group-housed females and their offspring, with sample sizes.

Measure		Pair-housed females				Group-housed females			
		Mean ± 1 SEM	n	Excluded	Missing sample	Mean ± 1 SEM	n	Excluded	Missing sample
Female weight, day 65		235.09 g	17 ♀♀	7 ♀♀		230.10 g	33 ♀♀	3 ♀♀	
		± 5.35				± 2.60	11 groups	1 group	
Female weight, day 87		243.85 g	13 ♀♀	11 ♀♀		241.67 g	12 ♀♀	24 ♀♀	
		± 6.64				± 6.41	4 groups	8 groups	
Age at first egg		45.48 days	23 ♀♀	1 ♀		45.47 days	36 ♀♀		
		± 0.74				± 0.57	12 groups		
Total nr of eggs collected		0.66 eggs/♀/day	361 eggs	1 ♀		0.60 eggs/♀/day	533 eggs		
		± 0.02	23 ♀♀			± 0.02	12 groups		
Egg mass		10.17 g	324 eggs	1 ♀		10.33 g	531 eggs		
		± 0.06	23 ♀♀			± 0.04	12 groups		
Stress protocol	Baseline	2.77 ng/ml	14 ♀♀	6 ♀♀	4 ♀♀	2.12 ng/ml	24 ♀♀	3 ♀♀	9 ♀♀
		± 0.22				± 0.14	11 groups		
	Post-challenge	13.05 ng/ml	14 ♀♀			11.05 ng/ml	22 ♀♀	1 group	11 ♀♀
		± 1.66				± 1.65	11 groups		
Yolk mass		2.78 g	51 eggs	6 ♀♀		2.78 g	73 eggs	6 ♀♀	
		± 0.04	18 ♀♀			± 0.03	10 groups	2 groups	
Yolk T	Concentration	5.02 pg/mg	17 eggs	6 ♀♀	1 ♀	5.81 pg/mg	24 eggs	6 ♀♀	
		± 0.89				± 0.86			
	Total	14.07 ng	17 ♀♀			15.86 ng	10 groups	2 groups	
		± 2.55				± 2.29			
GnRH challenge	Baseline	0.67 ng/ml	16 ♀♀	7 ♀♀	1 ♀	0.51 ng/ml	22 ♀♀	12 ♀♀	2 ♀♀
		± 0.05				± 0.03	8 groups		
	Post-challenge	0.72 ng/ml	16 ♀♀			0.56 ng/ml	22 ♀♀	4 groups	
		± 0.05				± 0.03	8 groups		
Eggs for F1	Eggs collected	6.89 eggs/♀	124 eggs	5 ♀♀	1 ♀^1^	6.46 eggs/♀	155 eggs	6 ♀♀	6 ♀♀^1^
		± 0.31				± 0.44			
	Eggs fertilized	6.06 eggs/♀				5.08 eggs/♀			
		± 0.30	18 ♀♀			± 0.55	8 groups	2 groups	
	Eggs hatched	3.72 eggs/♀				2.88 eggs/♀			
		± 0.46				± 0.52			
F1 offspring	Mass at hatching	7.18 g	66 chicks	5 ♀♀	2 ♀♀^1^^,^ ^2^	7.10 g	69 chicks	6 ♀♀	10 ♀♀^1^^,^ ^2^
		± 0.08				± 0.06			
	Tarsus at hatching	12.93 mm	17 ♀♀			12.77 mm	20 ♀♀	2 groups	
		± 0.06				± 0.07	8 groups		

^1^male infertile;

^2^females without offspring
