# Supplementary figures and images for: Social environment during egg laying: Changes in plasma hormones with no consequences for yolk hormones or fecundity in female Japanese quail, Coturnix japonica
Source: PLoS One. 2017 May 3;12(5):e0176146. doi: 10.1371/journal.pone.0176146 (PMC5414935; doi:10.1371/journal.pone.0176146)

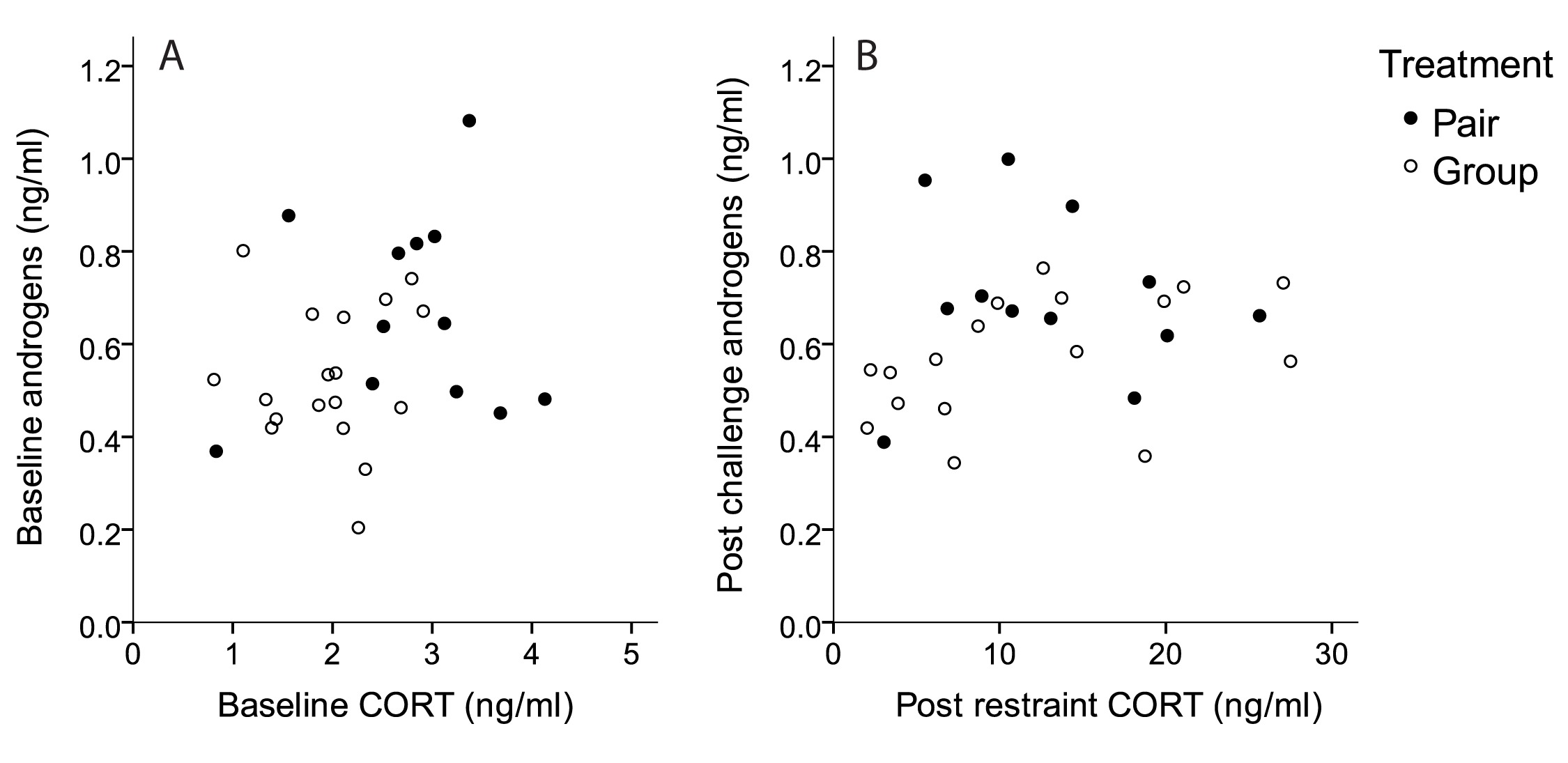

Supplement: S1 Fig — (A) Individual baseline plasma CORT concentrations plotted against baseline plasma androgen concentrations, and (B) individual post-challenge plasma CORT concentrations plotted against post-challenge androgen levels for both social treatments. Neither baseline nor post-challenge CORT and androgen concentrations were correlated with each other. (TIF) [file pone.0176146.s001.tif]

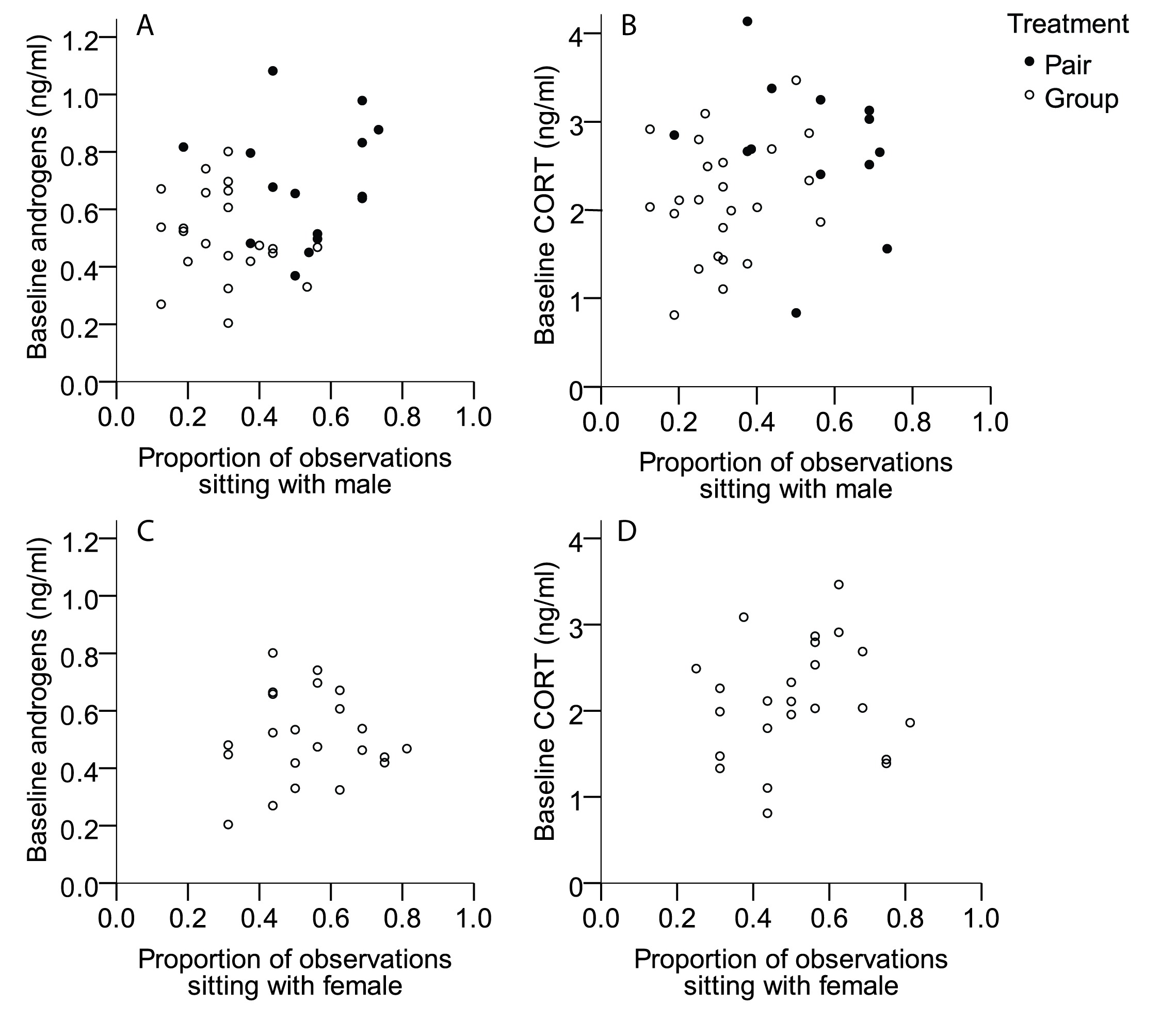

Supplement: S2 Fig — The proportion of all scan observations that a female was found sitting within a distance of one body-length from the male, plotted against the (A) baseline androgen concentration and the (B) baseline CORT concentration in her plasma, for both social treatments. The proportion of time a female spent sitting with the male did not predict baseline plasma CORT or androgen concentrations (CORT: F(1, 33.60) Sitting with male = 0.02, p = 0.88; androgens: F(1, 33.45) Sitting with male = 0.11, p = 0.75), and this was true for both social treatments (CORT: F(1, 32.95) Treatment*sitting with male = 1.32, p = 0.26; androgens: F(1, 32.40) Treatment*sitting with male = 0.45, p = 0.51). (C) and (D) show the proportion of scan observations that a group-housed female was found sitting close to another female, plotted against (C) her baseline plasma androgen concentration and (D) her baseline plasma CORT concentration. The proportion of time a female spent sitting with at least one other female did not predict baseline plasma CORT or androgen concentrations (CORT: F(1, 20.98) Sitting with female = 0.26, p = 0.62; androgens: F(1, 17.07) Sitting with female = 0.25, p = 0.63). (TIF) [file pone.0176146.s002.tif]

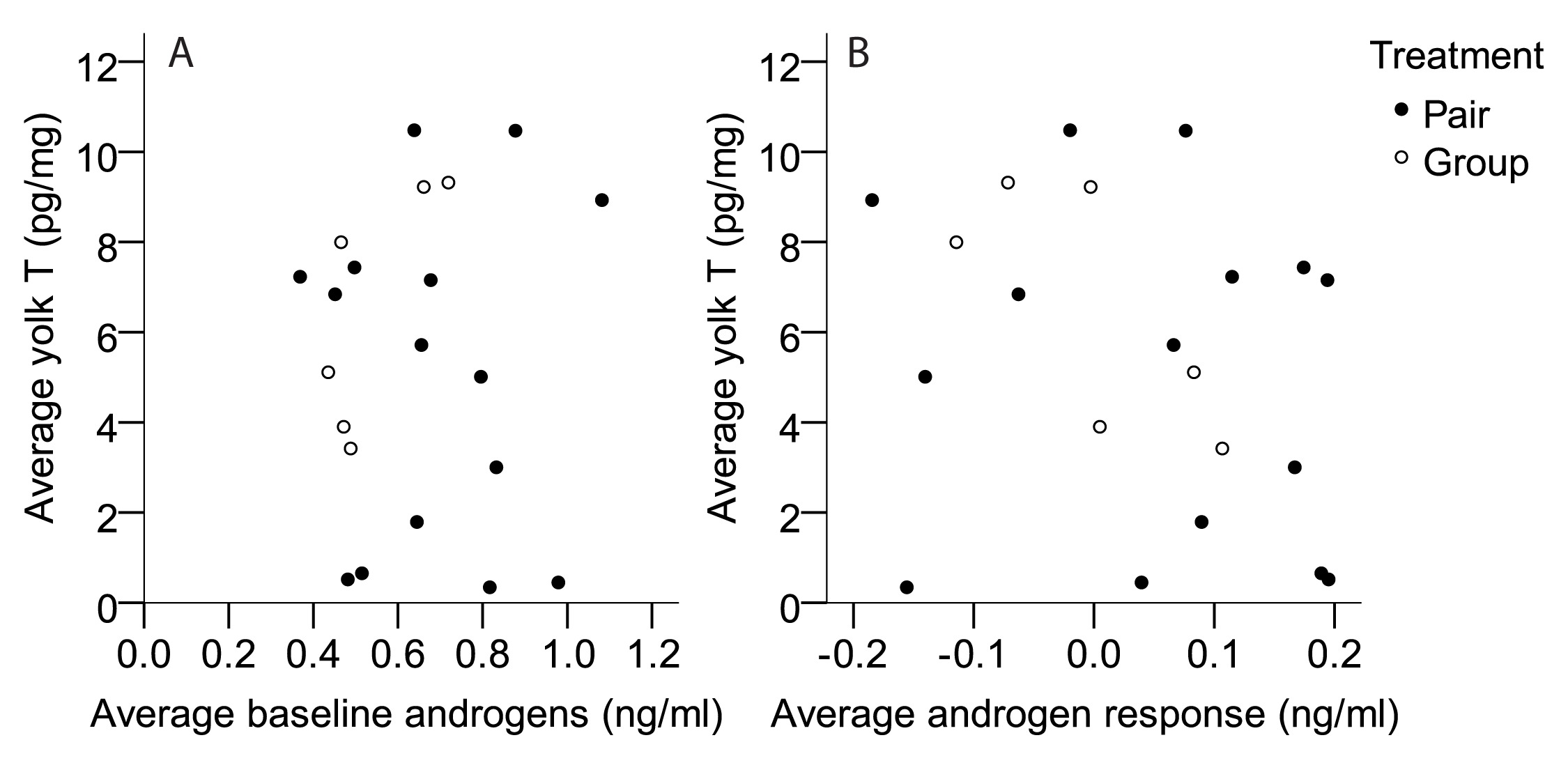

Supplement: S3 Fig — (A) Average baseline plasma androgen concentrations per cage and (B) the average androgen response to GnRH per cage plotted against the average yolk T concentrations per cage. Note that in pair housed-females, the average concentrations are the same as the individual female’s concentrations as there is only one female per cage. For group-housed females, hormone values were averaged per cage because eggs could not be assigned to individuals within groups. Neither baseline plasma androgen concentrations, nor the response to GnRH predicted yolk T concentrations. (TIF) [file pone.0176146.s003.tif]

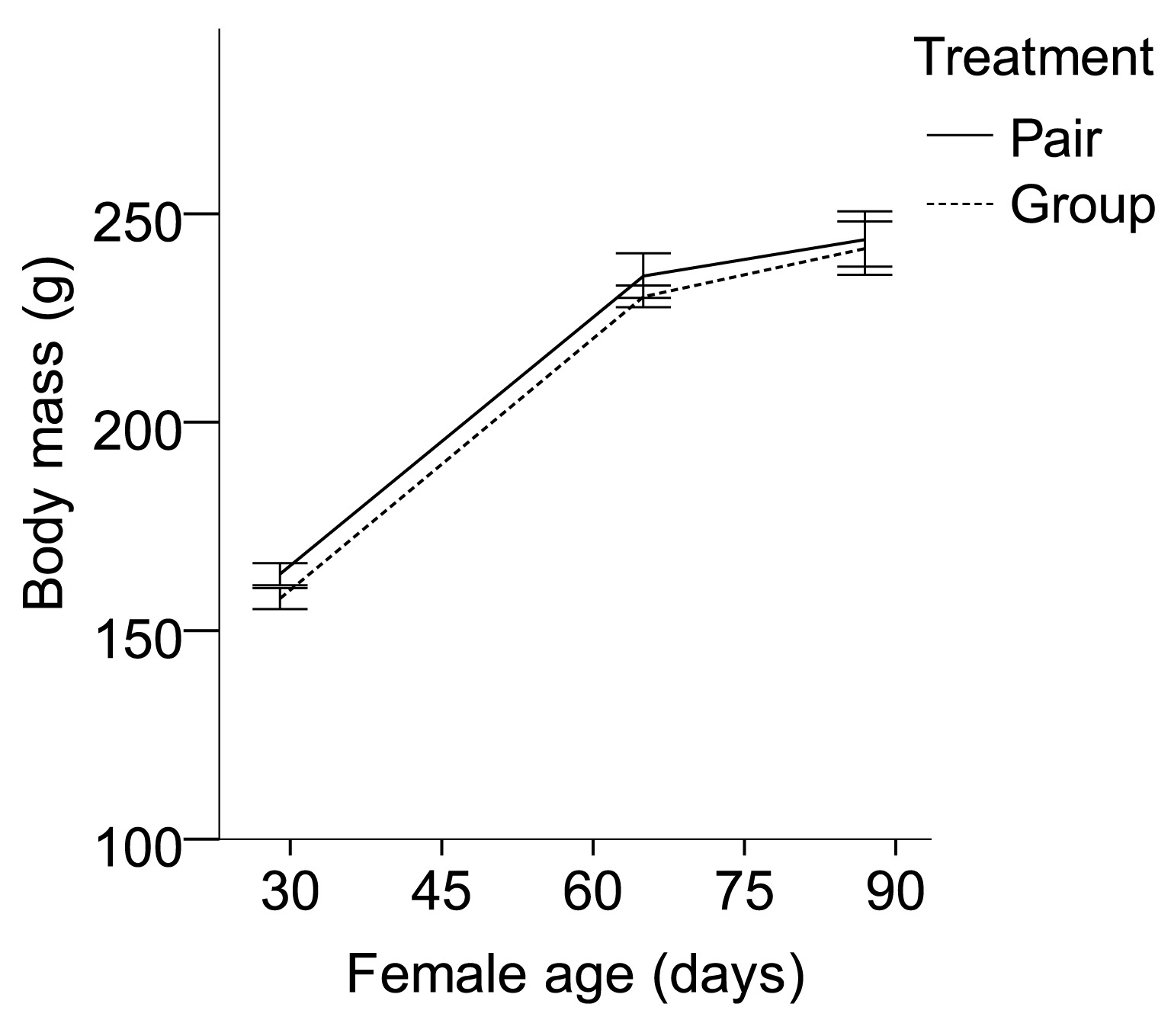

Supplement: S4 Fig — Depicted is the average body mass of both pair-housed and group-housed females, which did not differ between treatments, on day 19 (n = 24 pair-housed females and 36 group-housed females from 12 groups), 65 (n = 17 pair-housed females and 33 group-housed females from 11 groups) and day 87 (n = 13 pair-housed females and 12 group-housed females from 4 groups). Error bars indicate ± 1 SEM. (TIF) [file pone.0176146.s004.tif]
